# The Jan Sjödin faba bean mutant collection: morphological and molecular characterization

**DOI:** 10.1186/s41065-024-00339-7

**Published:** 2024-10-07

**Authors:** Hamid Khazaei, Ulrika Carlson-Nilsson, Alan H. Schulman

**Affiliations:** 1 Production systems, Natural Resources Institute Finland, Helsinki, Finland; 2 Department of Agricultural Sciences, University of Helsinki, Helsinki, Finland; 3 Nordic Genetic Resource Center, Alnarp, Sweden; 4 Institute of Biotechnology and Viikki Plant Science Centre, University of Helsinki, Helsinki, Finland

**Keywords:** *Vicia faba*, Mutant genetic resources, Genetic diversity, SPET genotyping

## Abstract

**Background:**

Plant mutagenesis creates novel alleles, thereby increasing genetic and phenotypic diversity. The availability of the faba bean (*Vicia faba* L.) reference genome and a growing set of additional genomic resources has increased the scientific and practical value of mutant collections. We aimed to genotype and morphologically phenotype a historical faba bean mutant collection developed and characterized by Jan Sjödin (1934–2023) over half a century ago in order to increase its value to researchers. The collection was genotyped using high-throughput single-primer enrichment technology (SPET) assays.

**Results:**

We used 11,073 informative single nucleotide polymorphism (SNP) markers spanning the faba bean genome to genotype 52 mutant lines along with the background line, cv. Primus. A range of flower, seed, leaf, and stipule mutations were observed. The analysis of population structure revealed a shallow structure with no major subpopulations. Principal component and cluster analyses revealed, to a minor extent, that the mutants clustered by their phenotype.

**Conclusions:**

The mutants’ phenotypic variation and shallow structure indicate that the Sjödin faba bean collection has the potential to play a significant role in faba bean breeding and in genetic and functional studies.

**Supplementary Information:**

The online version contains supplementary material available at 10.1186/s41065-024-00339-7.

## Background

Plant breeding relies heavily on diversity, both at the phenomic (trait diversity) and genomic (genetic diversity) levels. Heritable variation allows plant breeders to select superior genotypes, enabling the development of high-yielding and stress-resilient crops with improved quality traits. Plant mutagenesis is an important tool in plant breeding for creating random (or with gene editing, targeted) allelic diversity that was not present in the existing germplasm, enabling selection for traits otherwise not possible within the gene pool available to the breeder [1]. Mutagenesis is particularly important for crop species, such as faba bean (*Vicia faba* L.), having no known wild progenitor that could otherwise serve as a source of useful alleles for cultivar breeding [2]. The novel alleles derived from mutagenesis complement the existing diversity available in the germplasm collections of genebanks and in current breeding programs, facilitating progress in crop improvement in faba bean. Mutants are also highly useful for fundamental biological studies.

The potential of mutagenesis as a tool to increase diversity for faba bean improvement was first demonstrated by Jan Sjödin (4 April 1934–3 January 2023) in an extensive mutagenesis program run by the Swedish breeding company Svalöf Weibull AB. In this program, between 1960 and 1970, Sjödin developed a large collection of mutant lines in faba bean. He employed a range of physical and chemical mutagens, including X-ray, neutron, gamma-ray, ethyl methanesulfonate (EMS), methyl methanesulfonate, ethylene imine, 8-ethoxy-caffeine, and EMS plus Cu^++^, in the Svalöf faba bean “Primus” genetic background [3–5]. He collected, induced, classified, and crossed mutants and spontaneous variants displaying a wide variety of traits of interest [5]. He also used translocation lines derived from mutagenesis for the assignment of morphological markers (e.g., flower and seed coat color) to faba bean chromosomes [6–9]. The resulting lines were shared by him as valuable faba bean genetic resources for future breeding programs. Approximately 100 accessions of the J. Sjödin mutant collection were donated by Svalöf Weibull AB to NordGen (the Nordic Genetic Resource Center) in 2010 after the Forage Division at Svalöf Weibull was closed. The collection includes flower color, leaf, stipule, seed, and growth habit mutants. The collection is now conserved at NordGen.

About two decades after the work by J. Sjödin, other faba bean mutation collections were reported by researchers in Italy [10], Spain [11], Scotland [12], and France [13]. Various mutants in traits such as flower color, growth habit and leaf morphology, have been reported, particularly by the Italian and Spanish programs. In the last few years, several mutagenesis programs have been reported globally [e.g., 14, 15, 16, 17]. In faba bean, mutagenesis has been highly efficient in broadening the genetic basis for resistance to biotic stress, adaptation to abiotic stress, and for enhanced seed quality traits [18].

Despite their proliferation, the faba bean mutant collections have been subject primarily to morphological screening, with limited genomic characterization [19, Donal O’Sullivan, personal communication]. However, the recently developed faba bean genomic tools and resources [20–22] can efficiently accelerate molecular characterization of mutant collections, thereby accelerating faba bean improvement. Moreover, the recently released faba bean reference genome [22] has sped the design of single-primer enrichment technology (SPET) assays for high-throughput genotyping of this crop, which can be used to genetically characterize mutants. The main objectives of this study were to morphologically phenotype the J. Sjödin historical faba bean mutant collection and to use SPET genotyping to analyze its genetic diversity.

## Materials and methods

### Plant material

Eighty-five mutant lines from Sjödin’s faba bean collection, along with the background line cv. Primus, were obtained from NordGen (Alnarp, Sweden). Primus is a standard faba bean genotype characterized by wild-type spotted flowers (tannin-containing), buff seeds with a 1000-seed weight of approximately 400 g, compound leaves, and indeterminate inflorescence growth. Among the mutant lines, 11 failed to germinate. The accession numbers and mutant types are given in Table S1. The mutant lines were originally coded on the basis of their observable phenotypes: seed mutants (“Fr” stands for *frö*, Swedish for seed); flower mutants (“B” for *blomma*, Swedish for flower); stipule mutants (“St” for *stipel*, Swedish for stipule); unifoliata mutants (“U”, for unifoliate); the various other mutant types (“Ö”, for *övriga*, Swedish for others). The final accession list included 29 seed mutants, 21 flower mutants, 11 stipule mutants, three unifoliate mutants, and 10 other mutants, including two dwarfs and one of determinate growth habit (Table S1).

The mutant plants were evaluated in a climate-controlled greenhouse at the Department of Agricultural Sciences, University of Helsinki, Viikki campus, Finland. Seeds were inoculated with *Rhizobium leguminosarum* biovar. *viciae* (Elomestari Oy, Tornio, Finland) before sowing in 5 L plastic pots filled with peat (Taimiseos Professional W R8493, Kekkilä Oy, Vantaa, Finland). The pots were arranged in a randomized complete-block design with three replicates, each pot containing one plant. The photoperiod was 14 h light and 10 h dark; the temperature was maintained at 21 °C day/16°C night. The photosynthetic photon flux density was approximately 300 µmol m^− 2^ s^− 1^ at the canopy level, provided by high pressure sodium lamps. The relative humidity was maintained at 60%. The sowing date was 1 February 2023.

### Phenotyping for morphological characteristics

We recorded the observable morphological characteristics of flowers, seeds and stipules, as well as plant types, searching for phenotypes distinct from the wild type (cv. Primus). Every studied morphological trait was compared to the wild type. Faba bean flowers have a large standard petal, two smaller wing petals, and two fused keel petals. The wild-type flower has white petals with a black spot on each wing petal, often with dark vein markings on the standard petal. Faba bean flower colors may also be white (absence of pigments in the petals - zero tannin), yellow wing spots, brown, pink, diffuse yellow, or red [23]. The typical color of the faba bean seed coat (testa) is buff. It may also be purple (violet), red, zero tannin white (often turning gray), yellow, black, green, or brown [24, 25]. Faba bean leaves are usually compound pinnate, consisting of 2–7 leaflets. In the unifoliate mutant, a compound leaf is transformed into a simple leaf [4, 26]. Faba bean plants have stipules at the base of their leaves, which carry an extra-floral nectary marked in most genotypes by dark pigmentation [27]. The size of the stipules and pigmentation vary across faba bean genotypes.

A normal faba bean plant has indeterminate inflorescence growth, which typically ends when a plant reaches its maximum growth potential depending on its genotype and environment. A terminal inflorescence mutant reaches a terminal vegetative phase, after which the inflorescence transforms into a terminal floral meristem [5].

### DNA extraction and genotyping

Genomic DNA from 53 faba bean genotypes (Table S1) was extracted from fresh leaf tissue of single plants of greenhouse grown plants using a modified CTAB extraction method [28] Specific Primer Enrichment Technology (SPET) was used for genotyping [29]. SPET genotyping was performed at IGA Technology Services (IGATech, Udine, Italy). SPET library preparation and sequencing was carried out as described by Jayakodi et al. [22]. The results of genotyping revealed approximately 709 K single nucleotide polymorphisms (SNPs). The data were filtered to exclude entries with more than 5% missing data and 5% heterozygosity. A minor allele frequency (MAF) < 0.05 was applied.

### Population structure and genetic diversity

The “LEA” package in R was used to study population structure [30]. The sNMF function was used to run K (ancestral genetic groups) values from 1 to 10, with 100 repetitions. The LEA cross-validation method was used to identify the most likely K value. The population structure results were visualized via the R bar chart plot function. The SNP data were analyzed via TASSEL v5.2.15 [31] to calculate the MAF and polymorphic information content. The polymorphic information content (PIC) was calculated package “adegenet” in R [32]. Principal component analysis (PCA) was conducted via TASSEL 5.0. A dendrogram was generated from Nei’s distance [33] matrix via UPGMA (Unweighted Pair-Cluster Method using Arithmetic Averages), and the resulting tree was visualized with iTOL v6.9.1 [34]. The observed levels of heterozygosity were calculated in R via the “inbreedR” package [35].

## Results

### Phenotypic diversity

**Fig. 1:**
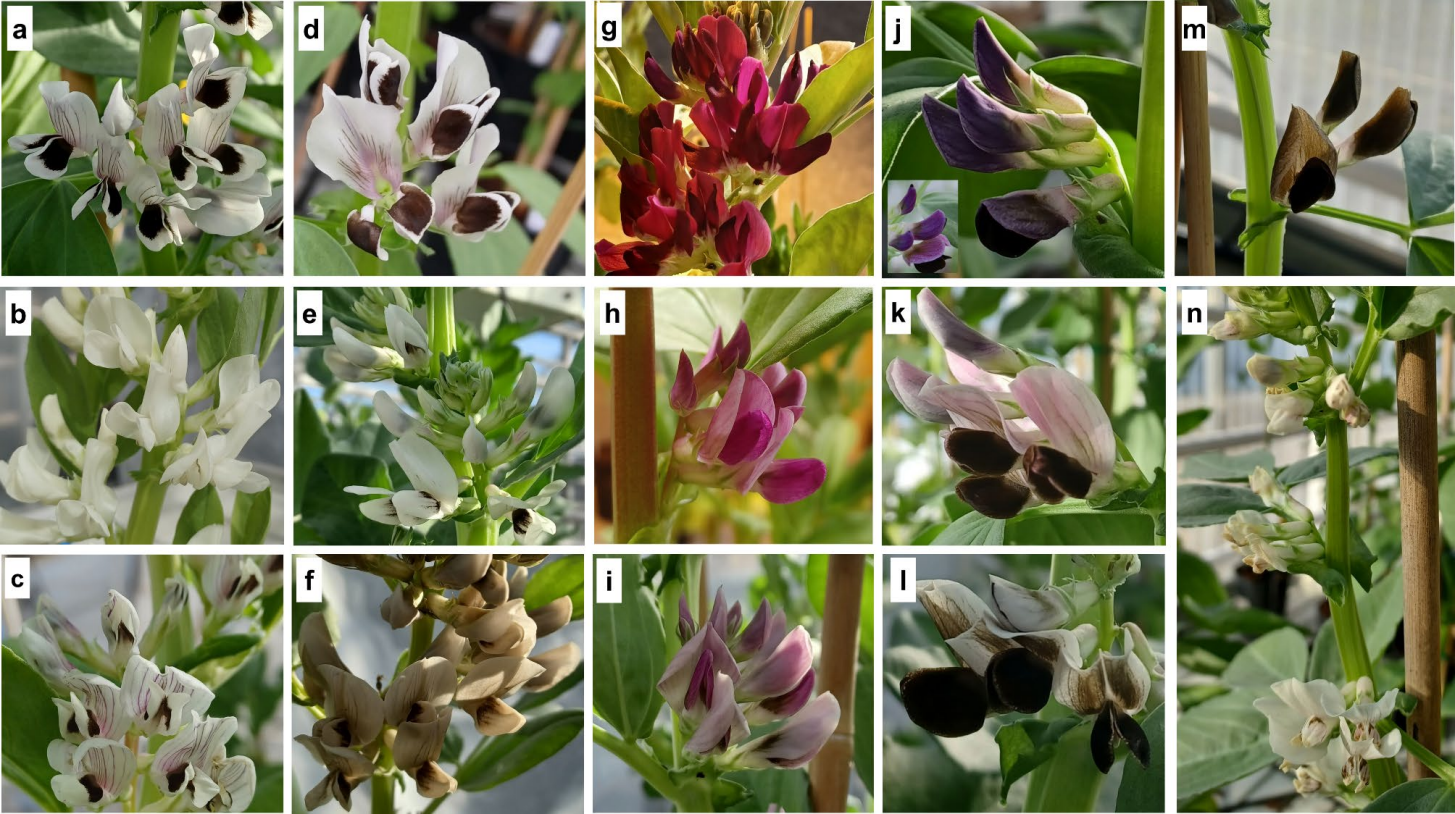
Flower color diversity in some Sjödin mutant lines. **(a)** cv. Primus (wild type). **(b)** St206 (white flower). **(c)** Fr 80 (presence of red veins on flower standard). **(d)** Fr6 (separated wing petals). **(e)** B42 (grayish brown flower). **(f)** St226 (faded wing spots). **(g)** cv. Karmesin (scarlet red-flowered, used as a check for colored flowers). **(h)** B10 (pink flower color). **(i)** B25 (pinkish standard and spotted wing petals). **(j)** B19 (violet flower color). **(k)** B8 (pink and brown flower color); **(l)** B37 (combinations of white and brown in the flower standard). **(m)** B17 (brown flower). **(n)** Fr3 (deformed flowers)

The observable characteristics of the faba bean flowers, mainly the standard and wing petals, are shown in Fig. 1 and described in Table S1. The cv. Primus and a red-flowered faba bean line (Karmesin) were used as color controls (Fig. 1a and g). As expected, all “B” mutant lines (flower mutants) produced colored flowers (Fig. 1f-m). Three “St” lines (stipule mutants) produced white flowers (e.g., Fig. 1b) and colorless stipule spots. Some of the “Fr” mutant lines (seed mutants) also produced off-type flowers, including pronounced red vein markings on the flower standard (Fig. 1c), separated wing petals (Fig. 1d), faded spots on wing petals (Fig. 1e), and deformed flowers (Fig. 1n). In flower mutant B42, the seedlings, calyx, pedicels, and pods also presented an olive color, which differentiates this mutant from the others (Figure S1).

**Fig. 2:**
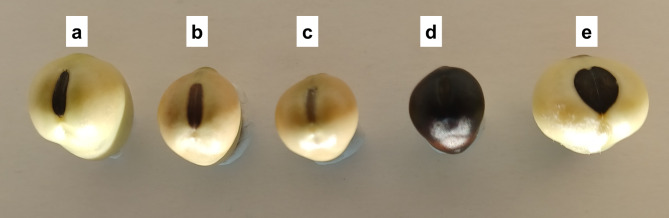
Hilum size and pigmentation diversity in some Sjödin mutant lines. **(a)** cv. Primus (wild type). **(b)** St232 (intermediate hilum color). **(c)** Ö53 (colorless hilum). **(d)** Fr9 (small hilum). **(e)** Fr41 (spherical hilum)

All the “B”, “St”, unifoliate (“U”) and other “Ö” mutant lines produced a wild-type buff seed coat color (Table S1). Seed coat color varied in the “Fr” seed mutant lines, including green, purple, red, brown, and black (Table S1). Some of the seed mutant lines are shown in Figure S1. The color and shape of the hilum also varied among the genotypes (Fig. 2). We were able to allocate some of the hilum mutant lines to the original reports by Sjödin (Fig. 2a-c). Seed mutant Fr41 corresponds to a spherical (round) hilum (*Hi-3*; Fig. 2e), while seed mutant Fr9 corresponds to a small hilum (either *Hi-1* or *Hi-2*; Fig. 2d).

**Fig. 3:**
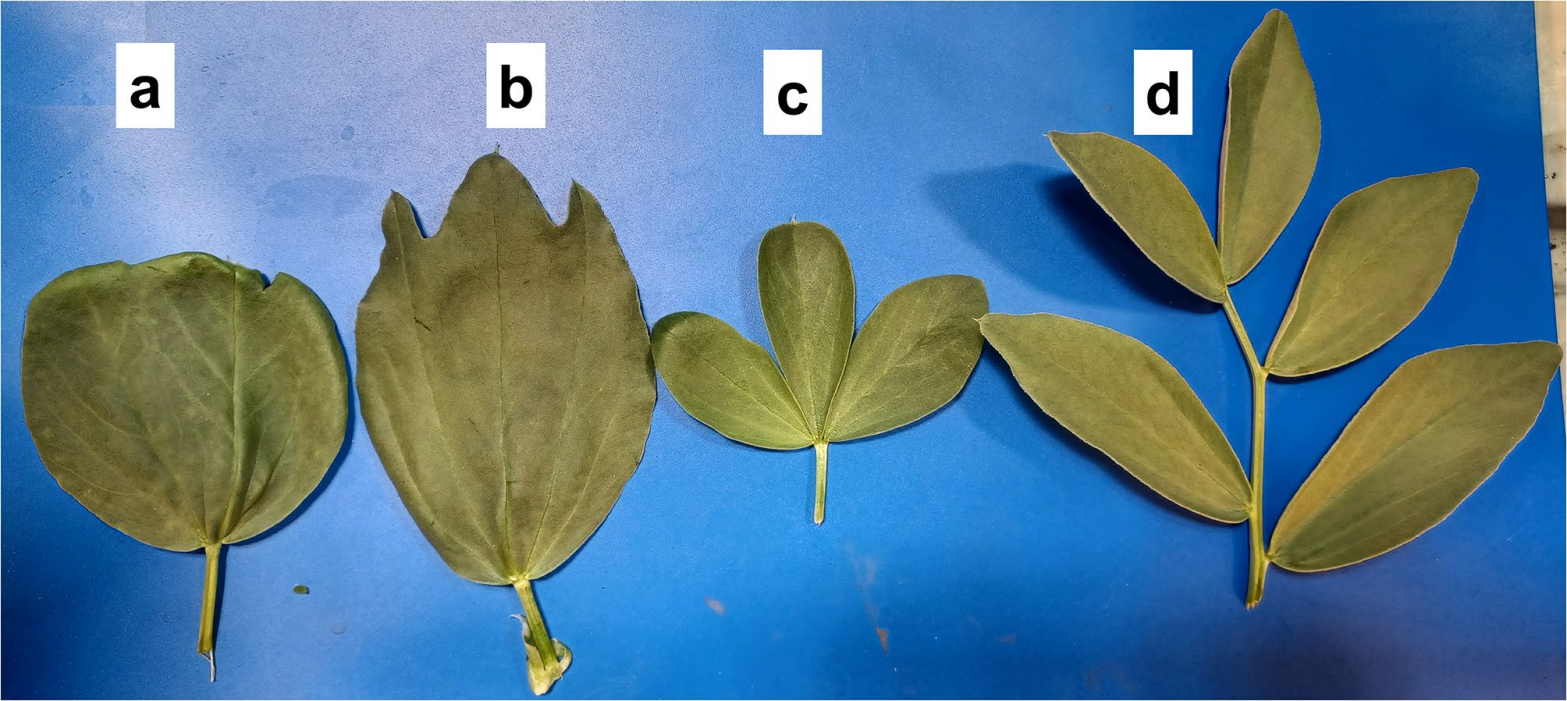
Leaf shape diversity in some Sjödin mutant lines. **(a)** U6 (unifoliate mutant). **(b)** U4 (attached trifoliate mutant). **(c)** U5 (trifoliate leaf mutant). **(d)** cv. Primus (wild type)

The leaf shape diversity is illustrated in Fig. 3, including a faba bean wild-type compound leaf (Fig. 3d). Unifoliate mutant lines U1, U6, and Ö41 are obligate unifoliate mutants (Fig. 3a); the unifoliate trait remains consistent throughout the entire vegetative period. U5 was a trifoliate leaf mutant (Fig. 3c); however, some leaves presented a bifoliate morphology. U4 differs from the other unifoliate mutants in that it has more triangular than round leaves, which are also split at the top (Fig. 3b). We also observed additional mutant lines, including dwarf (Ö53), terminal inflorescence (Ö38), and late ripening types (B27 and Fr19). More details on the morphological characterization of flowers, seeds, and other notable characteristics are presented in Table S1.

### Genomic diversity and population structure

Of the 709k markers, 11,073 high-quality polymorphic markers were retained after filtering. The highest number of SNP markers (2,939) was found on Chromosome 1, whereas Chromosome 6 had the lowest number of SNP markers (1,195). Chromosomes 2, 3, 4, and 5 contain 1,885, 1,740, 1,507, and 1,226 markers, respectively. Among the 11,073 markers, 581 were not assigned to chromosomes. The PIC value ranged from 0.099 to 0.500, with an average of 0.143. The mean minor-allele frequency ranged from 0.050 to 0.490, with an average of 0.079 (Table S2). The heterozygosity ranged from 0.03 to 0.21, with a mean of 0.06 in the studied mutation collection (Table S1).

**Fig. 4:**
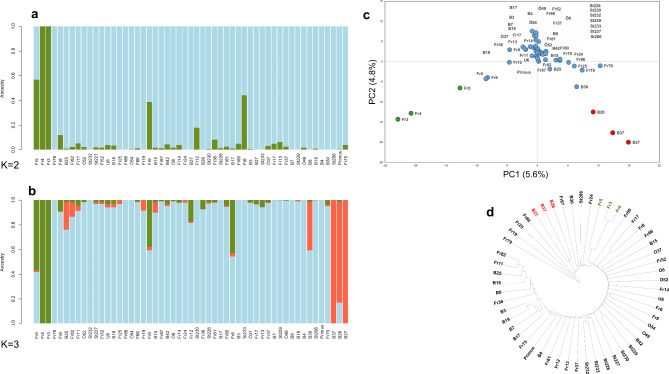
Genetic diversity of 52 Sjödin faba bean mutant lines along with their background line, cv. Primus. **a** and **b.** Admixture proportions, estimated by the LEA package at K2 and K3, are displayed in a bar plot. **c.** Principal component analysis of the 53 faba lines represented by the first two principal components (PC1 and PC2). **d.** UPGMA dendrogram based on genetic distance calculation showing relationships among faba bean genotypes

The sNMF structure analysis supported the presence of two main clusters (Figure S2). However, the most likely model (K = 2) admixture plot did not clearly show two groups (Fig. 4a). At K2, the seed mutant lines Fr3, Fr4, and Fr5 were grouped together. This suggests the presence of one population and a few outliers. K3 was added to one group consisting of three flower mutant lines (B26, B27, and B37) (Fig. 4b).

The PCA plot (Fig. 4c) revealed an inverted V-shaped pattern that was dispersed in the center, with a few outliers in the wings. The left wing consists of three seed mutant lines (Fr3, Fr4, and Fr5), whereas the right wing includes three flower mutant lines (B26, B27, and B37). The first two components explained more than 10% of the total variation. The PCA and clustering results (Fig. 4d) coincided, and both support the STRUCTURE results at K3.

## Discussion

Collections of mutants are familiar to communities working with model plant species (i.e., *Arabidopsis thaliana* and *Medicago truncatula*), while they are also transformative for upstream genetic studies and breeding in many crop species [*reviewed in*^36^]. Faba bean mutation collections are less well developed and characterized than those of many other crop species. With growing interest in this crop, owing to its protein-rich seeds and valuable ecological benefits, collections of mutants can play an important role by offering novel alleles for traits of interest. Recent advancements in faba bean genomics [22] have enhanced the design of high-throughput SPET markers, which were utilized to examine a historical mutation collection in this study. Several seed, flower, and leaf mutants have been observed, together with a shallow population structure.

We compared the morphological observations of the Sjödin collection with earlier described morphological variations in faba bean, including spontaneous and induced mutations [37], and with his original report [5]. Most of the mutant lines he had studied could be matched with existing mutants. Among those not earlier reported, the Fr6 flower mutant line produced also unique flowers with separated wing petals, resulting in a butterfly-like appearance. The U5 leaf mutant produced a mixture of bifoliate and trifoliate leaves, which likewise has not been previously reported. The mutant line St231 presented very high autofertility, with all the flowers setting seeds. To confirm these phenotypes, seeds harvested from single plants were re-evaluated under similar growing conditions in early 2024, with the same phenotypes being observed. These newly reported phenotypes, which were not originally reported by Sjödin [5] may occur during the population maintenance.

The mutation rate and population size may determine genetic diversity [38]. Our results revealed a shallow population structure in the studied population. To a minor extent, the observed diversity patterns reflect the mutant types, as some seed mutants (Fr3, Fr4, and Fr5) and flower mutants (B26, B27, and B37) clustered together. Low population structure was also observed in two other faba bean mutation populations [19]. We investigated only a small portion of the original Sjödin mutation population, which might be one of the reasons for the narrow genetic variability. Mutant line selection at early generations by Sjödin was likely focused on specific morphological traits (e.g., of flowers or seeds) leading to low genetic variability and population structure. The average heterozygosity rate was low (6%), suggesting limited outcrossing during population maintenance, which resulted in few opportunities for genetic recombination and maintained low population structure. This is important, as the population structure reflects the breeding history and pedigree of the population [39]. Notably, in partially outcrossing crop species such as faba bean, maintaining mutant individuals is laborious and requires either insect-proof facilities or sufficient isolation distances to maintain genetic purity.

Sjödin [5] identified two terminal inflorescence mutants, *ti-1* and *ti-2*, which significantly reduced the number of flowering nodes, resulting in the plant type being “topless”. He confirmed only the monogenic inheritance of *ti-1* [5]. We performed a few crosses between some of his mutant lines and other sources. One cross was made between the terminal inflorescence mutant (Ö38, Figure S1) and cv. Tina (F22_1143). Tina, which originated from Germany, also has a terminal inflorescence [40]. All F_1_ individuals from the cross Ö38 × Tina produced topless plants, suggesting that they carry the same *ti* gene. The terminal inflorescence is regulated by the *TERMINAL FLOWER1* (*TFL1*) gene [41]. *Vf_TFL1* is responsible for the determinate growth habit of faba bean [42], and it was shown that Ö38 carries this allele [43]. A cross between Ö53 (dwarf mutant) and a tall faba bean genotype was made to study the genetics of height in faba bean. At the time of writing, of the F_1_ seeds harvested, all have produced tall plants.

Recently, a SPET panel with 90,000 oligonucleotide probes was designed and used for genotyping 197 faba bean accessions [22]. In this study, we genotyped 53 faba bean genotypes from the Sjödin collection using the recently developed SPET SNP array, in which a set of 11,073 high-quality SNP markers were generated after rigid filtration. A recent study that characterized 128 Egyptian faba bean genotypes by SPET generated 6,759 SNP markers [44]. The Vfaba_v2 Axiom SNP array, which was applied to 2,678 faba bean genotypes representing global diversity, generated 21,345 SNP markers [45]. Furthermore, a 130 K targeted next-generation sequence-based genotyping platform on 410 faba bean accessions resulted in a total of 38,111 SNP markers [46]. Given this marker density, the newly developed genotyping arrays may be used for future genetic diversity and genome-wide association analysis in faba bean. SPET genotyping integrates the advantages of arrays and high-throughput sequencing technologies. SNP markers derived from SPET genotyping are highly enriched for the gene space, generating a set of gene-associated markers to be utilized for faba bean genetic studies. Our results suggest that SPET genotyping is a very effective approach for genotyping faba bean.

CRISPR/*Cas*9-mediated targeted mutagenesis—gene editing—has been successfully employed to develop new allelic variations in many crop species [*reviewed in*^47^], but it has not yet been applied to faba bean. This approach requires precise knowledge of the sequence of the target site, which is now available from the faba bean genome resources. However, the effective use of gene editing for faba bean improvement hinges on efficient transformation or regeneration, or both, for the target lines; this is currently the limiting step. Moreover, genome editing applications are restricted by legal constraints in some countries, including those of the European Union [48]. Consequently, induced random mutagenesis remains an important alternative. Faba bean-induced mutation collections, such as those of Sjödin, despite their limited population size, can still play a significant role in faba bean breeding and genetic studies.

## Conclusions

We linked a historical faba bean mutation collection with a newly developed SPET genotyping platform in order to recover the biological value embedded in the collection. Morphological observations revealed multiple flower, seed, leaf, and stipule mutants; however, limited population structure was observed. This study examined visible morphological traits, but deeper phenotyping, including for abiotic stress tolerance and biotic stress resistance, should be undertaken. We have secured funding to investigate the seed biochemistry of the Sjödin collection, with the aim of identifying novel sources of seed quality traits. Future studies genetic and genomics studies on the loci identified in the mutant lines reported here are needed.

## Electronic supplementary material

Below is the link to the electronic supplementary material.

**Supplementary Material 1**

**Supplementary Material 2**

**Supplementary Material 3**: **Figure S1. a.** B42 olive color pods. **b.** B42 flower color compared with that of the wild type (left) and white-flowered (right). **c.** Red (Fr80) and purple (Fr75) seed mutants. **d.** Terminal inflorescence mutant (Ö38) vs wild type (cv. Primus).

**Supplementary Material 4**: **Figure S2.** Cross-entropy values based on sNMF analysis for K = 1–10.

## Data Availability

The genotyping and phenotyping data presented in this study are available from the corresponding author upon request.

## References

[1] Sikora P, Chawade A, Larsson M, Olsson J, Olsson O. Mutagenesis as a tool in plant genetics, functional genomics, and breeding. Int J Plant Biol. 2011;2011:314829. 10.1155/2011/314829. 10.1155/2011/314829 PMC3270407 22315587

[2] Duc G, Bao S, Baum M, Redden B, Sadiki M, Suso MJ, Vishniakova M, Zong X. Diversity maintenance and use of *Vicia faba* L. genetic resources. Field Crops Res. 2010;115:270–8. 10.1016/j.fcr.2008.10.003.

[3] Sjödin J. Some observations in X1 and X2 of *Vicia faba* L., after treatment with different mutagens. Hereditas. 1962;48:565–86. https://www.cabidigitallibrary.org/doi/full/10.5555/19631603889.

[4] Sjödin J. Some unifoliata mutants in *Vicia faba* L. Hereditas. 1964;51:279–90. 10.1111/j.1601-5223.1964.tb01936.x.

[5] Sjödin J. Induced morphological variation in *Vicia faba* L. Hereditas. 1971;67:155–79. 10.1111/j.1601-5223.1971.tb02371.x.

[6] Sjödin J. (1965) Induced reciprocal translocations in *Vicia faba*. In: *Induction of Mutations and the Mutation Process* (Eds. J. Veleminsky and T. Gichner), Prague, Czech Republic, pp. 387–390. https://www.cabidigitallibrary.org/doi/full/10.5555/19671600124

[7] Sjödin J, Hagberg A. A survey of translocation studies in *Vicia faba* L. Hereditas. 1968;59:242–52. 10.1111/j.1601-5223.1968.tb02174.x.

[8] Sjödin J. Induced asynaptic mutants in *Vicia faba* L. Hereditas. 1970;66:215–32. 10.1111/j.1601-5223.1970.tb02347.x.

[9] Sjödin J. Induced paracentric and pericentric inversions in *Vicia faba* L. Hereditas. 1971;67:39–54. 10.1111/j.1601-5223.1971.tb02357.x.

[10] Filippetti A, De Pace C. Improvement of seed yield in *Vicia faba* L. by using experimental mutagenesis. II. Comparison of gamma-radiation and ethyl-methane-sulphonate (EMS) in production of morphological mutants. Euphytica. 1986;35:49–59. 10.1007/BF00028540.

[11] Cabrera A. Inheritance of flower color in *Vicia faba* L. FABIS. 1988;22:3–7.

[12] Ramsay G, Griffiths DW, Dow ND. Spontaneous and induced variation in levels of vicine and convicine in faba beans. Asp Appl Biol. 1991;27:43–7.

[13] Duc G. Mutagenesis of faba bean (*Vicia faba* L.) and the identification of five different genes controlling no nodulation, ineffective nodulation or supernodulation. Euphytica. 1995;83:147–52. 10.1007/BF01678042.

[14] O’Sullivan DM, Angra D. Advances in faba bean genetics and genomics. Front Genet. 2016;7:150. 10.3389/fgene.2016.00150. 27597858 10.3389/fgene.2016.00150 PMC4993074

[15] Khazaei H, Mäkelä PS, Stoddard FL. Ion beam irradiation mutagenesis in rye (*Secale cereale* L.), linseed (*Linum usitatissimum* L.) and faba bean (*Vicia faba* L.). Agri Food Sci. 2018;27:146–51. 10.23986/afsci.70780.

[16] Mao D, Michelmore S, Paull J, Preston C, Sutton T, Oldach K, Yang SY, McMurray L. Phenotypic and molecular characterisation of novel *Vicia faba* germplasm with tolerance to acetohydroxyacid synthase-inhibiting herbicides (AHAS) developed through mutagenesis techniques. Pest Manag Sci. 2019;75:2698–705. 10.1002/ps.5378. 30779284 10.1002/ps.5378

[17] Nurmansyah, Alghamdi SA, Migdadi HM. Morphological diversity of faba bean (*Vicia faba* L.) M_2_ mutant populations induced by gamma radiation and diethyl sulfate. J King Saud Univ Sci. 2020;32:1647–58. 10.1016/j.jksus.2019.12.024.

[18] Adhikari KN, Khazaei H, Ghaouti L, Maalouf F, Vandenberg A, Link W, et al. Conventional and molecular breeding tools for accelerating genetic gain in faba bean (*Vicia faba* L.). Front Plant Sci. 2021;12:744259. 10.3389/fpls.2021.744259. 10.3389/fpls.2021.744259 PMC8548637 34721470

[19] Nurmansyah, Migdadi HM, Alghamdi SS, Khan MA, Afzal M. Genetic diversity and population structure of two faba bean mutant populations based on AFLP markers. Legume Res. 2021;44:759–65. 10.18805/LR-594.

[20] Khazaei H, O’Sullivan DM, Stoddard FL, Adhikari KN, Paull JG, Schulman AH, Andersen SU, Vandenberg A. Recent advances in faba bean genetic and genomic tools for crop improvement. Legume Sci. 2021;3:e75. 10.1002/leg3.75. 10.1002/leg3.75 PMC8700193 34977588

[21] Auvinen P, Chang W, Holm L, Jääskeläinen M, Khazaei H, Laine PK, et al. (2023) A faba bean pan-genome for advancing sustainable protein security. Legume Perspect. 24:7–9. https://www.legumesociety.org/wp-content/uploads/2024/01/legum_perspect_24.pdf. Accessed 28 July 2024.

[22] Jayakodi M, Golicz AA, Kreplak J, Fechete LI, Angra D, et al. The giant diploid faba genome unlocks variation in a global protein crop. Nature. 2023;615:652–9. 10.1038/s41586-023-05791-5. 36890232 10.1038/s41586-023-05791-5 PMC10033403

[23] Hughes J, Khazaei H, Vandenberg A. The study of genetics of flower color in faba bean reveals generous diversity to be used in the horticulture industry. HortScience. 2020;55:1584–8. 10.21273/HORTSCI15238-20.

[24] Ricciardi L, Filippetti A, De Pace C, Marzano CF. Inheritance of seed coat colour in broad bean (*Vicia faba* L.). Euphytica. 1985;34:43–5. 10.1007/BF00022862.

[25] IBPGR/ICARDA. (1985) Faba bean descriptors. AGPG: IBPGR/85/116, Rome, Italy, p. 19. Accessed 28 July 2024. https://cgspace.cgiar.org/server/api/core/bitstreams/e5a81aac-90fc-4298-b22c-910fb25b7f30/content

[26] Suso MJ, Aguilar JA, Moreno MT. Registration of unifoliate faba bean genetic stocks. Crop Sci. 2003;43:1571–2. 10.2135/cropsci2003.1571.

[27] Khazaei H, O’Sullivan DM, Sillanpää MJ, Stoddard FL. Genetic analysis reveals a novel locus in *Vicia faba* decoupling pigmentation in the flower from that in the extra-floral nectaries. Mol Breed. 2014;34:1507–13. 10.1007/s11032-014-0100-9.

[28] Doyle JJ, Doyle JL. Isolation of plant DNA from fresh tissue. Focus. 1990;12:13–5.

[29] Barchi L, Acquadro A, Alonso D, Aprea G, Bassolino L, Demurtas O, Ferrante P, Gramazio P, Mini P, Portis E, Scaglione D, Toppino L, Vilanova S, Díez MJ, Rotino GL, Lanteri S, Prohens J, Giuliano G. Single Primer Enrichment Technology (SPET) for high-throughput genotyping in tomato and eggplant germplasm. Front Plant Sci. 2019;10:1005. 10.3389/fpls.2019.01005. 31440267 10.3389/fpls.2019.01005 PMC6693525

[30] Frichot E, François O. LEA: an R package for landscape and ecological association studies. Methods Ecol Evol. 2015;6:925–9. 10.1111/2041-210X.12382.

[31] Bradbury PJ, Zhang Z, Kroon DE, Casstevens TM, Ramdoss Y, Buckler ES. TASSEL: software for association mapping of complex traits in diverse samples. Bioinformatics. 2007;23:2633–5. 10.1093/bioinformatics/btm308. 17586829 10.1093/bioinformatics/btm308

[32] Jombart T. *adegenet*: a R package for the multivariate analysis of genetic markers. Bioinformatics. 2008;24:1403–5. 10.1093/bioinformatics/btn129. 18397895 10.1093/bioinformatics/btn129

[33] Nei M. Analysis of gene diversity in subdivided populations. Proc Natl Acad Sci USA. 1973;70:3321–3. 10.1073/pnas.70.12.3321. 4519626 10.1073/pnas.70.12.3321 PMC427228

[34] Letunic I, Bork P. Interactive tree of life (iTOL) v6: recent updates to the phylogenetic tree display and annotation tool. Nucleic Acids Res. 2024;52:W78–82. 10.1093/nar/gkae268. 38613393 10.1093/nar/gkae268 PMC11223838

[35] Stoffel MA, Esser M, Kardos M, Humble E, Nichols H, David P, Hoffman JI. *inbrandR*: An R package for the analysis of inbreeding based on genetic markers. Methods Ecol Evol. 2016;7:1331–9. 10.1111/2041-210X.12588.

[36] Prasanna S, Jain SM. Mutant resources and mutagenomics in crop plants. Emir J Food Agric. 2017;29:651–7. 10.9755/ejfa.2017.v29.i9.86.

[37] ICARDA - International Center for Agricultural Research in the Dry Areas. Third conspectus of genetic variation within *Vicia faba*. Syria: FABIS, Aleppo; 1986. p. 54.

[38] Xu S, Stapley J, Gablenz S, Boyer J, Appenroth KJ, Sree KS, Gershenzon J, Widmer A, Huber M. Low genetic variation is associated with low mutation rate in the giant duckweed. Nat Commun. 2019;10:1243. 10.1038/s41467-019-09235-5. 30886148 10.1038/s41467-019-09235-5 PMC6423293

[39] Sim S-C, Robbins M, Deynze AV, Michel AP, Francis DM. Population structure and genetic differentiation associated with breeding history and selection in tomato (*Solanum lycopersicum* L.). Heredity. 2011;106:927–35. 10.1038/hdy.2010.139. 10.1038/hdy.2010.139 PMC3186243 21081965

[40] Link W, Hanafy M, Malenica N, Jacobsen H-J, Jelenić S. Faba bean. In: Kole C, Hall TC, editors. Compendium of transgenic crop plants: transgenic legume grains and forages. New York: Wiley; 2008; vol. 3, p. 71–88.

[41] Benlloch R, Berbel A, Ali L, Gohari G, Millán T, Madueño F. Genetic control of inflorescence architecture in legumes. Front Plant Sci. 2015;6:543. 10.3389/fpls.2015.00543. 26257753 10.3389/fpls.2015.00543 PMC4508509

[42] Avila CM, Atienza SG, Moreno MT, Torres AM. Development of a new diagnostic marker for growth habit selection in faba bean (*Vicia faba* L.) breeding. Theor Appl Genet. 2007;115:1075. 10.1007/s00122-007-0633-y. 17828523 10.1007/s00122-007-0633-y

[43] Östberg J. (2021) *Vicia faba* determinate and indeterminate inflorescence genotypes – comparison of genetic variation at *TFL1* locus. In: Independent Project in Biology: Swedish University of Agricultural Sciences, Alnarp, Sweden, p. 64. https://stud.epsilon.slu.se/16453/1/Ostberg_J_210215.pdf. Accessed 28 July 2024.

[44] Sallam A, Amro A, Mourad AMI, Rafeek A, Boerner A, Eltaher S. Molecular genetic diversity and linkage disequilibrium structure of the Egyptian faba bean using single primer enrichment technology (SPET). BMC Genom. 2024;25:644. 10.1186/s12864-024-10245-x. 10.1186/s12864-024-10245-x PMC11212244 38943067

[45] Skovbjerg CK, Angra D, Robertson-Shersby-Harvie T, Kreplak J, Ecke W, Windhorst A, Kærgaard Nielsen L, Schiemann A, Knudsen J, Gutierrez N, Tagkouli V, Fechete LI, Janss L, Stougaard J, Warsame A, Alves S, Khazaei H, Link W, Torres AM, O’Sullivan DM, Andersen SU. Genetic analysis of global faba bean diversity, agronomic traits and selection signatures. Theor Appl Genet. 2023;136:114. 10.1007/s00122-023-04360-8. 37074596 10.1007/s00122-023-04360-8 PMC10115707

[46] Zhang H, Liu Y, Zong X, Teng C, Hou W, Li P, Du D. Genetic diversity of global faba bean germplasm resources based on the 130K TNGS genotyping platform. Agronomy. 2023;13:811. 10.3390/agronomy13030811.

[47] Bhuyan SJ, Kumar M, Ramrao Devde P, Rai AC, Mishra AK, Singh PK, Siddique KHM. Progress in gene editing tools, implications and success in plants: a review. Front Genome Edit. 2023;5:1272678. 10.3389/fgeed.2023.1272678. 10.3389/fgeed.2023.1272678 PMC10744593 38144710

[48] Schulman AH, Oksman-Caldentey K-M, Teeri TH. European Court of Justice delivers no justice to Europe on genome-edited crops. Plant Biotechnol J. 2020;18:8–10. 10.1111/pbi.13200. 31246337 10.1111/pbi.13200 PMC6920151

