# Supplementary figures and images for: The Jan Sjödin faba bean mutant collection: morphological and molecular characterization
Source: Hereditas. 2024 Oct 7;161:37. doi: 10.1186/s41065-024-00339-7 (PMC11457391; doi:10.1186/s41065-024-00339-7)

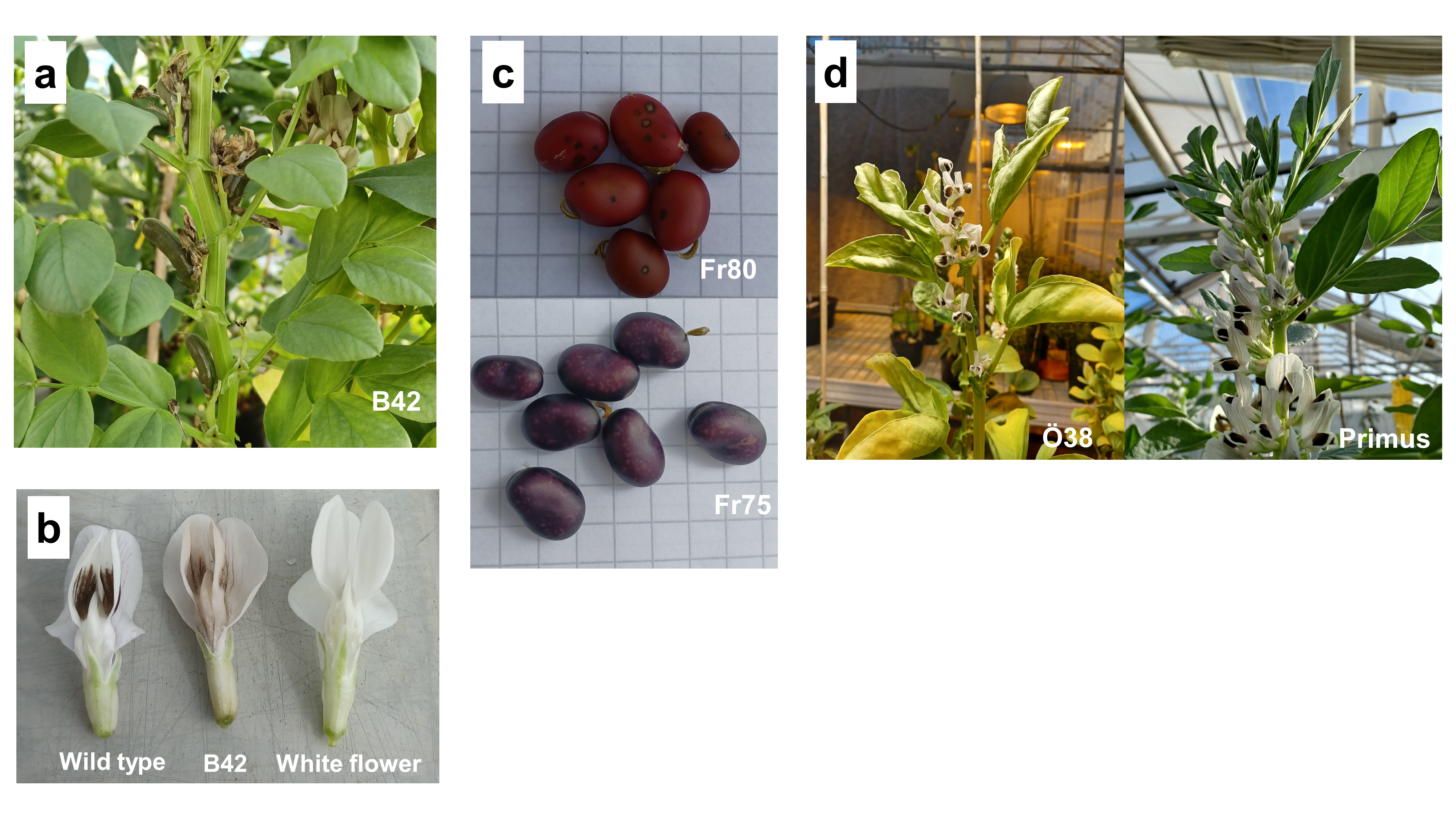

Supplement: Supplementary file 3 — Supplementary Material 3: Figure S1. a. B42 olive color pods. b. B42 flower color compared with that of the wild type (left) and white-flowered (right). c. Red (Fr80) and purple (Fr75) seed mutants. d. Terminal inflorescence mutant (Ö38) vs wild type (cv. Primus). [file 41065_2024_339_MOESM3_ESM.tif]

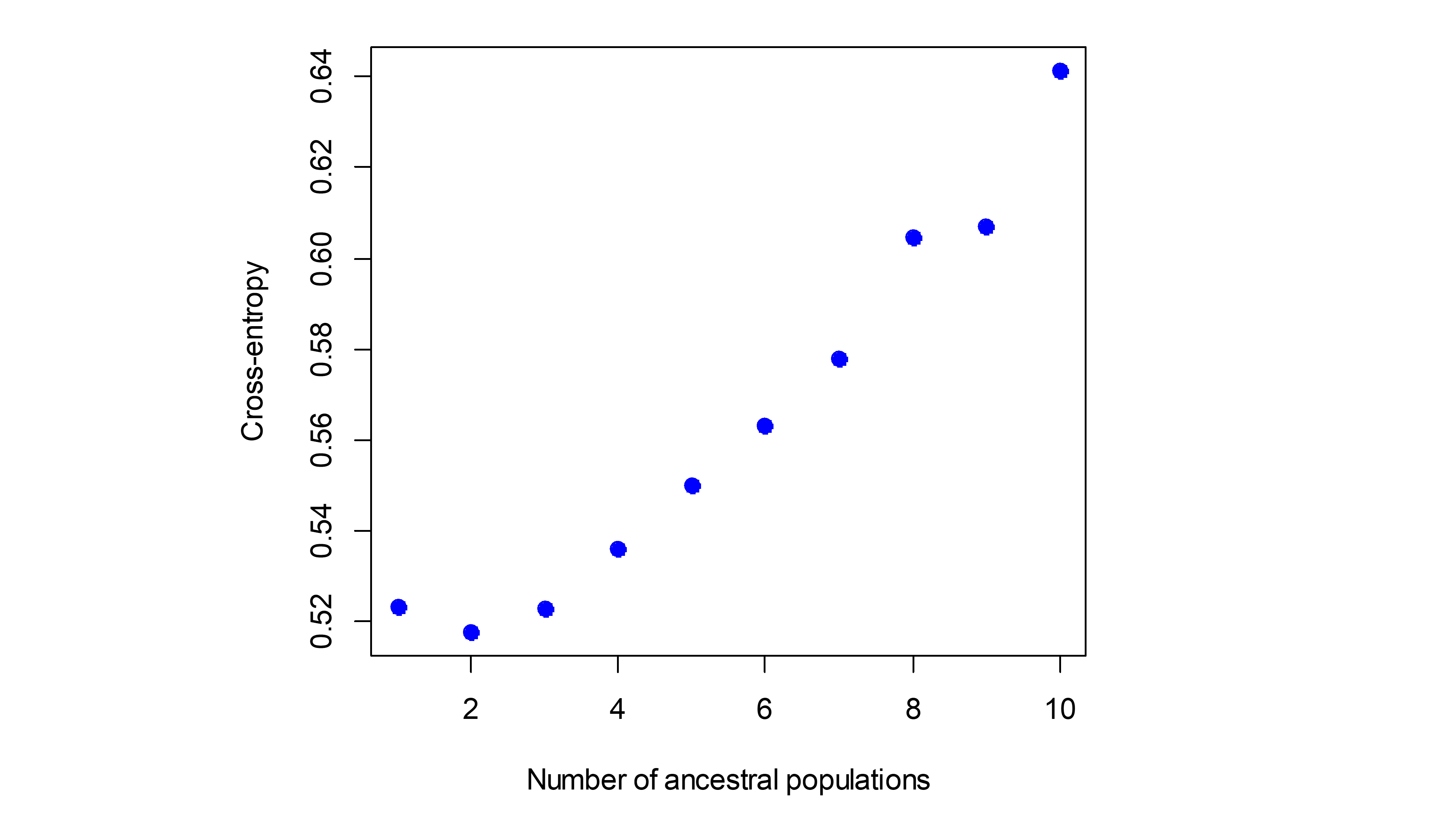

Supplement: Supplementary file 4 — Supplementary Material 4: Figure S2. Cross-entropy values based on sNMF analysis for K = 1–10. [file 41065_2024_339_MOESM4_ESM.tif]
